# Calibration uncertainty in molecular dating analyses: there is no substitute for the prior evaluation of time priors

**DOI:** 10.1098/rspb.2014.1013

**Published:** 2015-01-07

**Authors:** Rachel C. M. Warnock, James F. Parham, Walter G. Joyce, Tyler R. Lyson, Philip C. J. Donoghue

**Affiliations:** 1 School of Earth Sciences, University of Bristol, Bristol, UK; 2 National Evolutionary Synthesis Center, Durham, NC, USA; 3 Department of Paleobiology, Smithsonian Institution, Washington DC, USA; 4 John D. Cooper Archaeological and Paleontological Center, Department of Geological Sciences, California State University, Fullerton, CA, USA; 5 Department of Geosciences, University of Fribourg/Freiburg, Switzerland; 6 Denver Museum of Nature and Science, Denver, CO, USA

**Keywords:** molecular clock, calibration, cross-validation, fossil record, Bayesian, priors

## Abstract

Calibration is the rate-determining step in every molecular clock analysis and, hence, considerable effort has been expended in the development of approaches to distinguish good from bad calibrations. These can be categorized into *a priori* evaluation of the intrinsic fossil evidence, and *a posteriori* evaluation of congruence through cross-validation. We contrasted these competing approaches and explored the impact of different interpretations of the fossil evidence upon Bayesian divergence time estimation. The results demonstrate that *a posteriori* approaches can lead to the selection of erroneous calibrations. Bayesian posterior estimates are also shown to be extremely sensitive to the probabilistic interpretation of temporal constraints. Furthermore, the effective time priors implemented within an analysis differ for individual calibrations when employed alone and in differing combination with others. This compromises the implicit assumption of all calibration consistency methods, that the impact of an individual calibration is the same when used alone or in unison with others. Thus, the most effective means of establishing the quality of fossil-based calibrations is through *a priori* evaluation of the intrinsic palaeontological, stratigraphic, geochronological and phylogenetic data. However, effort expended in establishing calibrations will not be rewarded unless they are implemented faithfully in divergence time analyses.

## 1. Introduction

The molecular clock uniquely combines evidence from both molecular sequences and palaeontological and geological temporal constraints on sequence divergence, to establish evolutionary timescales. However, the precision of divergence time estimates is often so broad that they do not provide for effective tests of evolutionary hypotheses. It has been demonstrated that there is a modest limit on the gains in precision that can be obtained with increasing sequence data, beyond which increased precision can be obtained only by increasing the precision of fossil calibrations [1–3]. Unfortunately, identifying calibrations that are merely accurate is difficult enough. Two principal (but not necessarily mutually exclusive) approaches have emerged: (i) the *a priori* assessment of the empirical fossil anatomical, phylogenetic, stratigraphic and geochronological evidence, versus (ii) the *a posteriori* evaluation of the consistency of calibrations within a set.

*A priori* best practice requires that fossil calibrations comprise a conservative minimum constraint on a clade's age, minimizing phylogenetic uncertainty. In converting this into a calibration, the approach most widely adopted is to assign a non-uniform probability density (e.g. lognormal, exponential), fixed on the minimum constraint, that expresses a generalized view of the degree to which minima approximate divergence dates [3–12], invariably established without justification [13]. Alternatively, qualitatively justified ‘soft maxima’ have been established on palaeontological and geological grounds, based on the absence of evidence for a lineage antedating its oldest fossil record, qualified by the presence of taphonomic controls provided by sister lineages [6] (cf. [14–16]), and known gaps and facies biases in the rock record [15,17]. Effectively, the *a priori* establishment of minimum and maximum constraints based on fossil evidence removes them from equivocation. However, in practice, this approach is necessarily conservative in the identification of fossil taxa suitable for use in calibration, and in interpreting their age, such that calibrations established in this way are often a poor, or imprecise, approximation of divergence dates.

Alternatively, *a posteriori* methods have been developed to assess the relative quality of calibrations through the consistency with which each calibration, within a set, estimates the others when used in isolation. The underlying assumption is that calibrations should be consistent and inconsistent calibrations should be rejected. This approach has also been used to consider competing phylogenetic positions for critical fossils [18–23]. In attempting to address phylogenetic and stratigraphic uncertainty, Marshall [24] established, and Dornburg *et al*. [25] extended, a method for selecting the fossil calibration(s) among a set that provide the best approximation of the antiquity of the respective lineage(s). *A posteriori* methods keep fossil data at arm's length, assessing internal consistency or its veracity measured with respect to branch length.

We set out to evaluate the performance *a posteriori* versus *a priori* approaches for assessing calibration quality. Our analyses are based on the now classic empirical dataset that encompasses extant turtle phylogeny used to first demonstrate a number of *a posteriori* methods of evaluating calibration quality (e.g. [24–26]) and in debate about the importance of establishing calibration quality *a priori* [27–29]. We also employ a completely revised set of calibrations for this phylogeny, constructed following the principles of best practice [30]. These exemplify the impact of the *a priori* evaluation of fossil calibrations and, as such, they can be considered accurate, if not precise. Since debate over calibration quality has not considered seriously the impact of different approaches for establishing maximum constraints*,* we first explore the impact of different approaches to constraining node ages. We simulated the approach of assigning a non-uniform probability density to fossil-based minimum constraints, and contrast these results to those of analyses in which a uniform density is employed. We employed the cross-validation method of Near *et al*. [26,31] to measure consistency among calibrations based on minimum constraints, and adopted a novel cross-validation approach considering the entire timespan between minimum and maximum constraints [32].

Crucially, our results demonstrate that: (i) *a posteriori* methods have led to the recurrent selection of erroneous constraints, and (ii) the effective time priors implemented in an analysis differ for individual calibrations when employed alone or in variable combinations with additional constraints—this means that estimates of calibration quality based on consistency do not provide a faithful indication of how a given calibration will impact the analysis in combination with others. *A posteriori* approaches to assessing calibration quality cannot therefore substitute for the *a priori* evaluation of fossil evidence in establishing accurate constraints. However, the accuracy of any calibration may be compromised by the way in which the calibrations are effectively implemented in the Bayesian estimation of divergence times.

## 2. Material and methods

### (a) Modelling non-uniform and uniform priors using fossils

Bayesian molecular clock analyses were performed using the approximate likelihood approach implemented in MCMCTree [2,3,33], because it is computationally efficient [34] and uses a more predictable procedure in the construction of the joint time prior, in comparison to BEAST [3,35]. However, we reproduced our analyses in BEAST 1.6.1 [8,36] using uniform priors, to explore differences in the construction of the joint time prior. Fossil-based minimum and maximum constraints were established for this dataset following best practice [27,30].

Non-uniform priors express approximations of divergence timing relative to a minimum constraint, however, such calibrations are rarely evidence-based [13]. Although there are objective approaches to informing non-uniform prior densities (e.g. [37]), the turtle fossil record has not yet been documented in a manner that would allow time priors to be established in this way. By contrast, uniform priors allow the user to accommodate a view that nothing is known about the time of divergence relative to the constraints. We present this as a null hypothesis—that given the absence of evidence to the contrary, there is an equal prior probability of the timing of the divergence event, per unit time, spanning the minimum and maximum bounds; this is not an uninformative prior. We implemented hard minima, such that the probability that a divergence time postdates the minimum constraint approximates zero. Where applicable, we implemented soft maxima, allowing 2.5% of the probability to exceed maximum constraints [3].

We explored the use of non-uniform calibration priors, permuting the truncated Cauchy distribution, to reflect variable non-uniform probabilities of divergence timing relative to the minimum constraints [12]. A maximum bound must be specified at the root of the tree and so we retained a uniform distribution at the root, corresponding to the fossil-based calibration available for the age of crown turtles [27]. All molecular clock analyses were performed without sequence data to examine the effective priors, compared to the specified priors.

### (b) *A posteriori* evaluation of calibration quality

We implemented the original cross-validation method described in [26] to compare the consistency between our calibrations. Consistency was assessed: (i) relative to minimum constraints only, and (ii) relative to minimum and maximum constraints [32]. For each individual calibration, during each round of cross-validation, the tree was calibrated using a single uniform calibration prior, with a hard minimum and soft maximum constraint based on fossil evidence [27]. A soft maximum age constraint was applied at the root using the fossil-based maximum for the age of this node.

Finally, we compared three *a posteriori* approaches to evaluating calibrations [24–26] to the *a priori* evaluation of fossil evidence. To assess the quality of calibrations selected using *a posteriori* methods, we contrasted the selection of calibrations based on assessments of calibration quality among the Testudines dataset used in the seminal application of the cross-validation method (and its derivatives) [24–26]. This was compared to the *a priori* assessment of calibration quality based on the intrinsic palaeontological evidence used to establish these constraints, which formed the basis of an independent study [27]. Further details of all materials and methods are provided in the electronic supplementary material.

## 3. Results

### (a) The impact of non-uniform and uniform calibration priors

**Figure 1.**
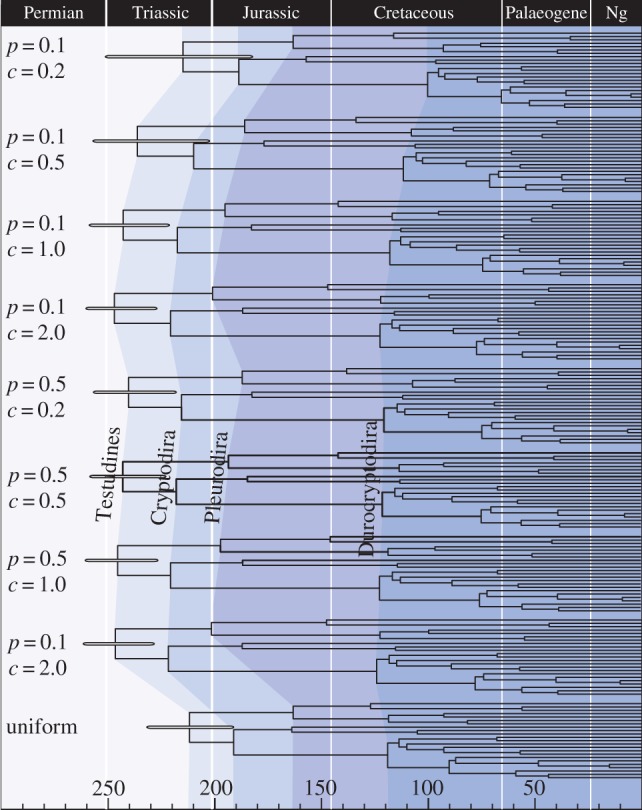
The posterior mean estimates obtained when the truncated Cauchy distribution was used to approximate the time of divergence relative to fossil-based minima in MCMCTree. Results are shown for two values of the location parameter *p* (0.1, 0.5) and four values of the scale parameter *c* (0.1, 0.5, 1, 2) and results are contrasted to those obtained when a uniform distribution is used to constrain node ages between the fossil-based minima and maxima. The branching order (and corresponding node labels) is the same as those shown in figure 3. The branch lengths represent the posterior means of the node ages. Four nodes are connected across the analyses to facilitate comparison. The 95% higher posterior density is indicated at the root of the tree. Ages are presented in millions of years before present. (Online version in colour.)

Increasing the uncertainty in the timing of divergence relative to fossil minima, based on a non-uniform prior, led to an increase in both prior and posterior age estimates across all nodes (figure 1; electronic supplementary material, table S1). Increasing uncertainty also generated more diffuse credibility intervals, except at the root (node 1: Testudines). The mean root age increased from 215 to 246 Ma, but the 95% intervals were attenuated with increasing uncertainty at the internal nodes. The results appear to be influenced strongly by the limit on the root: the posterior estimates appear to become only as ancient as the soft maximum at the base of the tree will allow (figure 1); note that MCMCTree requires the user to specify a soft maximum constraint at the root of the tree. In BEAST, if the upper (soft) constraint is not specified, then this limit will be specified indirectly by other parameters. The results obtained using uniform priors are different to those obtained using non-uniform priors, including the proposed temporal sequence of non-hierarchically dependent divergence events (figure 1; electronic supplementary material, table S1). For example, the posterior confidence intervals obtained using uniform priors suggest that of the two major groups of turtles, Cryptodira (node 8) originated before Pleurodira (node 2). By contrast, there is substantial overlap between the estimates obtained using non-uniform priors for the age of these nodes and, consequently, a coincident time of origin cannot be rejected.

### (b) *A posteriori* evaluation of calibration quality

**Figure 2.**
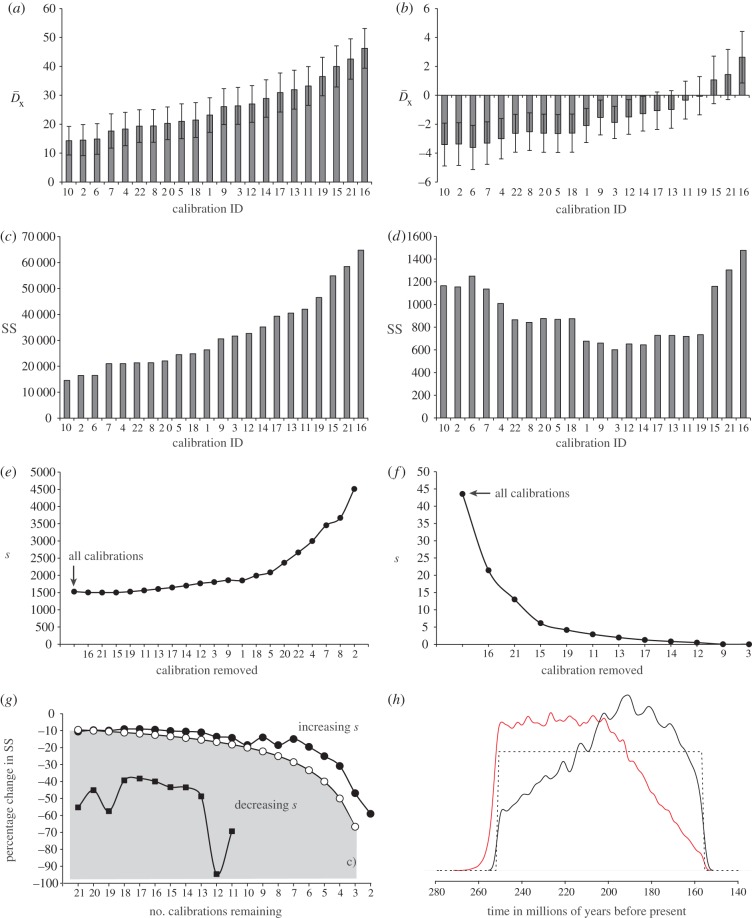
(*a*–*d*) Histograms showing the average difference (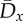) (*a*,*b*) and the sum of squared differences (SS) (*c*,*d*) between molecular- and fossil-based estimates obtained during each round of cross-validation. The values obtained when fossil-based minima are used to calculate 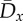 (*a*) and SS (*c*), and the values obtained when both minima and maxima are considered in the estimation of 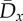 (*b*) and SS (*d*). The error bars shown in (*a*,*b*) reflect the standard error of the mean. (*e*–*f*) Plots showing the impact of sequentially removing calibrations on the average squared deviation(s) between molecular—and fossil—estimates considering minima only (*e*) and both minima and maxima (*f*). Sequential removal of the most inconsistent calibrations revealed a consistent, albeit statistically non-significant increase in *s* when only the fossil-based minima are used in the estimation of SS (*f*). By contrast, there is a steady albeit statistically non-significant decline in *s* when minimum and maximum temporal constraints are used to calculate SS (*e*). The value of *s* will increase if the rate of change of the denominator, *n*(*n* – 1) in the equation used to calculate *s*, exceeds the rate of change of the numerator ∑SS, where *n* represents the total number of calibrations [38]. (*g*) Illustrates the percentage change in ∑SS with the sequential removal of calibrations, estimated using minima (black circles) or both minima and maxima (black squares). This is contrasted to the rate of change of *n*(*n* – 1), where *n* represents to total number of calibrations used to calculate *s* (open circles). The rate of change of *n*(*n* – 1) always exceeds the rate of change of ∑SS when fossil minima are used in the calculation, hence we observe an increase in *s.* The converse is true when both minimum and maximum constraints are incorporated into the estimation of SS. Changes in *s* fluctuate markedly with the removal of calibrations as the value of *s* approaches zero; SS = 0 with the inclusion of the final 10 calibrations. (*h*) The effect of truncation in the establishment of the joint time prior at the root (node 1, figure 3) in BEAST (black) and MCMCTree (red). The dashed line represents the user-specified uniform prior. (*a*–*g*) All values were estimated and are presented in terms of millions of years. (Online version in colour.)

The results of the cross-validation analysis showed that the most consistent calibrations based on fossil minima are the most inconsistent calibrations based on minima and maxima; these are the nodes that tend to produce the youngest average estimates (figure 2*a–d*). Conversely, the most inconsistent calibrations based on fossil minima tend to produce the oldest average estimates; these nodes are the most frequently underestimated and tend to overestimate the age at other nodes. The results of the cross-validation analysis, which considered the minimum constraints only, are presented in figure 2*a*,*c*. The 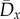 and the SS values for each calibration reflect the average differences between the mean molecular estimates and the minimum constraints of all other nodes. All 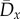 values are positive and range from 14.29 to 46.24 Myr, indicating that most posterior estimates of divergence times do not postdate fossil minima.

The results of the cross-validation analysis, in which both minimum and maximum age constraints are considered, are presented in figure 2*b*,*d*. The 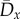 and the sum of squared differences (SS) values for each calibration reflect the average differences between the mean estimates and the minimum *or* maximum constraints of all other nodes. Most 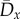 values are slightly negative and range from 3.60 to 2.63 Myr. SS values are two orders of magnitude smaller than those based on minimum constraints alone (figure 2*c,d*). This reflects the use of conservative maximum age constraints and the informative maximum limit placed at the root (251.4 Ma), which precludes estimates from becoming unjustifiably ancient. Few molecular estimates are likely to exceed their respective maxima. Regardless of the direction or magnitude of inconsistency, cross-validation analyses demonstrate that independent calibrations produce appreciably different divergence estimates. However, the removal of any calibrations did not significantly reduce the variance among calibrations and molecular estimates (figure 2*e*–*g*).

A comparison between three *a posteriori* approaches to assessing calibration quality shows that different numerical methods of defining calibration quality identify variable suites of constraints as either accurate or inaccurate (table 1). The calibrations selected for rejection using alternative *a posteriori* methods are neither supported by each other, nor by the available fossil evidence on which the constraints are based. The *a priori* evaluation of palaeontological evidence led to a ubiquitous, and in some cases substantial (up to 89%), revision of the minimum (and maximum) age constraints for all nodes. Although *a posteriori* approaches correctly discriminate some of the constraints that were necessarily revised based on fossil evidence, they also eliminated a number of accurate constraints, while retaining a number of inaccurate constraints (table 1). This means that *a posteriori* methods can lead to the selection of calibrations that are not supported by available fossil evidence.

**Table 1.** *A posteriori* versus *a priori* approaches to assessing calibration quality. Dash symbols denote n.a.

node^a^	divergence	fossil^b^	minimum^c^	maximum^d^	*a posteriori* assessment			*a priori* assess. (minimum)^e^	minimum	maximum
					Near *et al.* [26]	Marshall [24]	Dornburg *et al.* [25]	Joyce *et al.* [27]		
1 (1)	Testudines	*Proterochersis robusta*	210_F/F/M_	—	consistent	—	omitted *a priori*	phyl. misplaced	155.6	251.4
2 (3)	Pleurodira	*Araripemys barretoi*	110_F−M/F/M_	134.4	consistent	—	consistent	accurate minima	111	165.2
3 (4)	Pelomedusoides	*Cearachelys placidoi*	110_F–M/F/M_	134.4	consistent	—	consistent	accurate minima	92.8	149.5
4 (14)	Pelomedusidae	*Pelusios rusingae*	18_F/F/M_	22.0	consistent	—	inconsistent	inaccurately dated	5.3	149.5
5 (11)	Chelidae	*Yaminuechelys gasparinii*	71_F/F/M_	86.7	inconsistent	—	consistent	accurate minima	65.2	149.5
6 (15)	Chelodininae	*Chelodina* sp. and *Elseya* sp.	15_F/F/M_	18.3	inconsistent	—	inconsistent	accurate minima	11.6	149.5
7 (16)	*Chelus*–*Phrynops*	*Chelus* sp.	11.6_F/F/M_	14.2	inconsistent	—	inconsistent	accurate minima	13.4	149.5
8 (2)	Cryptodira	*Sandownia harrisi*	110_F–M/F/M_	—	consistent	—	omitted *a priori*	phyl. misplaced	124	200.2
10 (6)	Trionychidae	*Aspideretes maortuensis*	100_F–M/F/M_	122.1	consistent	selected for calibration	consistent	phyl. untested	17.3	149.5
13 (5)	Chelonioidea	*Santanachelys gaffneyi*	110_F/F/M_	134.3	inconsistent	inconsistent	consistent/ inconsistent	phyl. misplaced	48.4	149.5
15 (7)	Kinosternoidea	*Hoplochelys* sp.	65_F/F/M_	79.4	consistent	—	consistent	accurate minima, alternative fossils now available	70	149.5
16 (10)	Kinosternidae	*Baltemys* sp.	50_F/F/M_	61.1	consistent	—	consistent	accurate minima	52.8	149.5
19 (12)	Emydidae	*Chrysemys antiqua*	34_F/F/M_	41.5	consistent	—	consistent	accurate minima	32	100.5
20 (17)	*Graptemys*–*Trachemys*	*Trachemys inflata*	5_F/F/M_	6.1	inconsistent	—	inconsistent	phyl. untested	3	34
21 (8)	Testuguria	*Hadrianus majusculus*	52_F/F/M_	63.5	consistent	—	consistent	accurate minima	50.3	100.5
22 (9)	*Heosemys*–*Mauremys*	“*Ocadia*” *crassa*	50_F/F/M_	61.1	inconsistent	—	consistent	phyl. untested	5.3	65.8
— (13)	—	Lindholmemydidae	90_F/F/M_	109.9	consistent	—	consistent	phyl. untested	–	–
9 (18)	Trionychia	—	—	—	—	—	—	—	124	177.6
11 (19)	Durocryptodira	—	—	—	—	—	—	—	88.6	149.5
12 (20)	Americhelydia	—	—	—	—	—	—	—	70	149.5
14 (21)	Chelydroidea	—	—	—	—	—	—	—	70	149.5
17 (—)	Testudinoidea	—	—	—	—	—	—	—	50.3	149.5
18 (—)	Emydidae–*Platysternon*	—	—	—	—	—	—	—	32	100.5

^a^Node ID corresponds to those shown in figure 3. Numbers in brackets refer to the corresponding ID in Near *et al*. [26].

^b^Fossil specimens used to assess calibration quality *a posteriori*.

^c^Minimum (M) or fixed (F) age constraint used to assess calibration quality *a posteriori* in Near *et al*. [26], Marshall [24] and Dornburg *et al*. [25].

^d^Soft maximum age constraint used to assess calibration quality *a posteriori* in Dornburg *et al*. [25]. Maxima established using Marshall [24]; eqn. (11).

^e^*A priori* assessment of minimum fossil constraints only. Assessments indicate whether fossil specimens have been phylogenetically (phy.) misplaced or untested, inaccurately dated or provide appropriate minimum constraints.

### (c) Effective versus user-specified calibration priors

Analysis without sequence data demonstrates that the specified calibration priors are not faithfully implemented in the joint estimation of divergence times. This occurs in association with both non-uniform and uniform probability distributions. In the case of non-uniform time priors, increasing the uncertainty associated with the calibrations produced more imprecise specified time priors, but the effective marginal densities still do not match the specified time prior. This change, between the specified and the effective priors, is particularly significant at the root where the uniform specified prior is transformed into a distinctly non-uniform effective prior. This had a large impact on the prior credibility intervals for the root. The upper (maximum) 95% prior interval at the root always exceeded the specified soft maximum (251.5 Ma) by up to 5 Myr, but the lower (minimum) 95% prior interval became older (up to 55 Myr) than the specified minimum (155.6 Ma) (electronic supplementary material, table S1).

**Figure 3.**
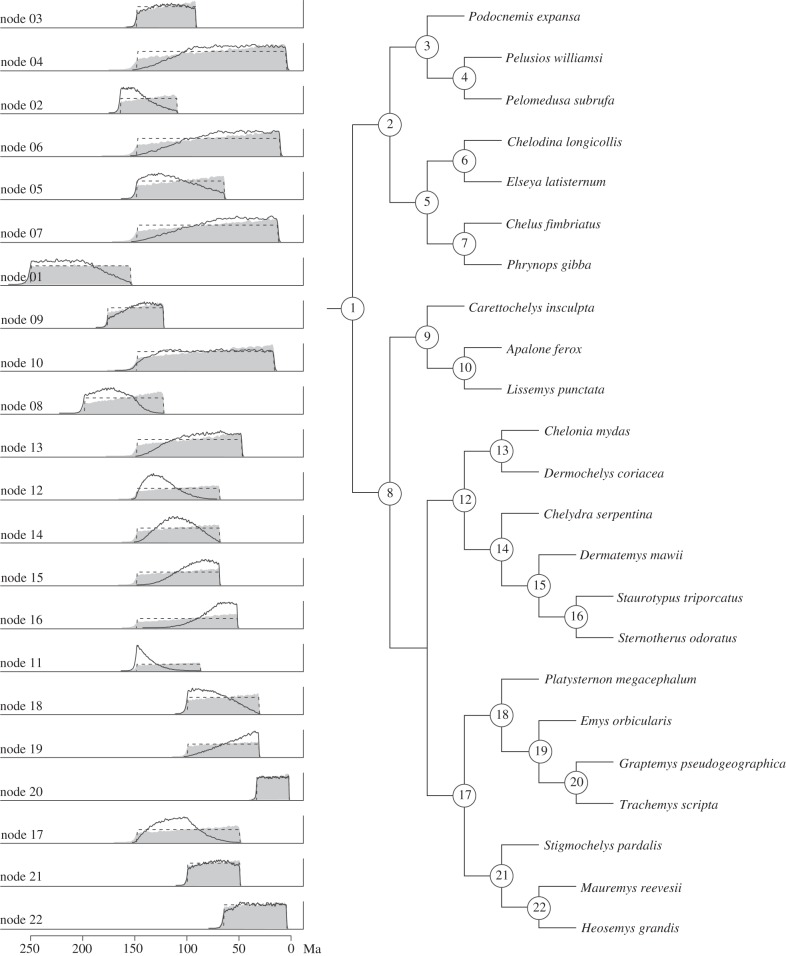
Plots contrasting the user-specified uniform calibration priors (dashed lines), firstly, with the effective marginal priors observed when each node is used for calibration during independent rounds of cross-validation (grey plots) and secondly, with the effective priors observed when all calibrations are combined in the final analysis in MCMCTree (black lines). This diagram illustrates how the interaction between different calibrations in the joint prior can result in effective priors that deviate substantially from the initial user-specified distributions.

We compared the specified uniform age priors in MCMCTree to: (i) the effective priors for each node during independent rounds of cross-validation, and (ii) the effective priors observed at each node when all calibrations are combined in a single analysis (figure 3). Even during cross-validation, when a single uniform calibration is employed, the marginal calibration densities do not always match the specified uniform densities. The largest discrepancies between the specified and effective priors are associated with nodes that have the broadest calibration spans and, hence, overlap most with the specified constraints on ancestral nodes (e.g. node 4: Pelomedusidae).

### (d) Comparison between BEAST and MCMCTree

**Figure 4.**
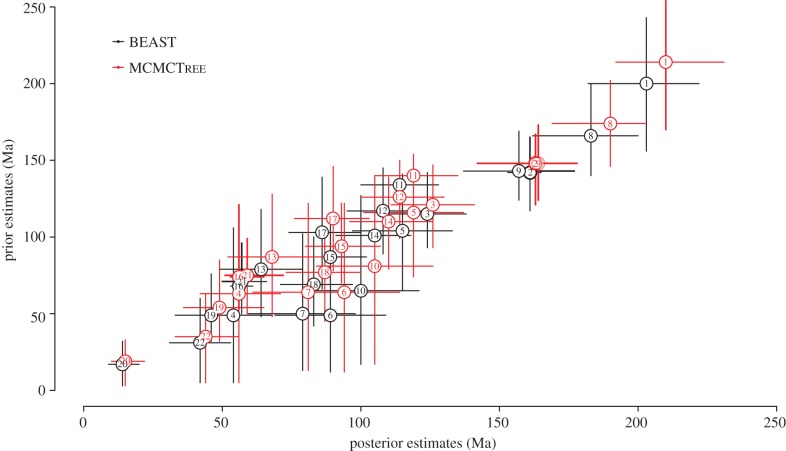
Plot contrasting the prior and posterior estimates (mean and 95% credibility intervals) obtained using BEAST (black) and MCMCTree (red). Node labels correspond to those shown in figure 3. (Online version in colour.)

BEAST and MCMCTree derived similar prior and posterior estimates of divergence times (figure 4), though MCMCTree produced slightly older mean estimates and wider credibility intervals. The largest difference was observed in estimates of root age. When all calibrations are combined in a single analysis, the effective prior densities obtained using BEAST and MCMCTree are similar and exhibit the same direction of skew and modality—with the main exception of the root (node 1: Testudines; figure 2*h*). The effective root age prior implemented in MCMCTree indicates that an older time of divergence is more likely. Conversely, in BEAST, the effective root age prior suggests that younger divergence times are more likely.

## 4. Discussion

### (a) The impact of non-uniform and uniform calibration priors

In the absence of fossil-based maximum constraints, the specified uncertainty associated with constraints may be made subjectively large (or small). Estimates of divergence times are evidently sensitive to the parameters used to specify the prior density. In figure 1, we contrast the posteriors obtained using non-uniform priors, to those obtained using uniform fossil-based minima and soft maxima where each node is constrained using a uniform prior. The comparison shows that these analyses yield very different results, including differences in the relative temporal sequence, not merely absolute timing, of speciation events.

These results corroborate the findings of previous studies [12,13,32], demonstrating that subtle changes in the parameters that describe the priors have an adverse impact upon the posterior divergence time estimates. Since there is frequently no material basis for selecting among the parameters, or the distributions that they control, in the majority of studies the time priors are, quite literally, unjustified [13]. Note that if no alternate evidence exists, relying on the default settings for the calibration priors in BEAST or MCMCTree is equivalent to accepting them and their implicit assumptions about the time of divergence relative to the constraints. For instance, if a soft maximum is not defined explicitly, this constraint will be specified indirectly by other parameters. Alternative approaches to informing calibration priors—for example, those based on stratigraphic occurrence data (e.g. [37,39])—represent an exciting area of development. However, these methods require large, well-curated fossil databases that are rarely available and, therefore, implemented.

### (b) The impact of specified and effective calibration priors

In all contemporary Bayesian molecular clock programs, the initial specified calibration priors will not be the same as the effective calibration priors actually implemented in the estimation of divergence times [12,13,30,32,35]. This is because the specified calibrations are truncated in the construction of the joint prior on times, to satisfy the expectation that ancestral nodes are older than descendent nodes [3,40]. Truncation is particularly obvious, given multiple overlapping constraints (e.g. [13]). However, even if there is no temporal or topological overlap between a pair of calibrations, their interaction with the tree prior can still result in differences in the effective priors (figure 3).

BEAST and MCMCTree differ in the way they construct the joint prior on times. Effectively, the same palaeontological constraints are implemented as different time priors by these two principal software packages. In BEAST, the specified and effective priors can be very different, even when only a single calibration is employed. This is because BEAST uses a multiplicative construction, by multiplying the calibration densities by the tree prior, which is necessary for the estimation of topology [35]. This can sometimes cause the effective calibration priors to violate the palaeontological constraints, and it is difficult to predict the impact of including multiple constraints [35].

Differences between the models that underlie BEAST and MCMCTree manifest themselves in both the prior and posterior estimates of divergence times. In particular, we have demonstrated that the same fossil constraints will lead to different effective time priors. The largest difference between BEAST and MCMCTree posterior estimates was observed at the root and is probably a direct consequence of differences in modality observed in the specification of the root age prior (figure 2*h*). Since the posteriors are sensitive to different time priors, as evidenced by the impact of variable non-uniform and uniform priors, this has material consequences for posterior molecular clock estimates. It is clear is that the effort expended in establishing accurate palaeontological time priors will not be repaid unless they are reflected in the effective time priors. The specified priors should be permuted experimentally until the primary palaeontological constraints are reflected in the effective time prior.

### (c) *A posteriori* versus *a priori* approaches to assessing calibration quality

Time priors have a substantial impact upon the outcome of divergence time analyses, and so it is necessary to discriminate between ‘good’ and ‘bad’ calibrations. Hence, there has been a great deal of effort expended in establishing criteria on which fossil calibrations should be based [5–7,13,30,32,41,42], and in developing methodological approaches to discriminating misleading fossil calibrations [18–26,31,38,43,44]. The *a posteriori* original cross-validation approach [26,31] and its subsequent developments [22,24,25,38] emphasize calibration consistency as the most desirable quality in a set of calibrations. The underlying assumptions of the cross-validation approach to assessing calibration quality have been criticized previously [5,24]. The results of our analyses identify two additional and ultimately fatal problems with the cross-validation approach: (i) cross-validation methods demonstrably result in the selection of calibrations that are not supported by the available fossil evidence (table 1), and (ii) this approach is compromised within the Bayesian framework because the effective priors for a given calibration vary depending on the presence or the absence of other constraints (figure 3). This final point is particularly problematic since it demonstrates violation of the basic implicit assumption of cross-validation methods, that individual calibrations perform in the same manner regardless of whether they are employed individually or in combination with other calibrations.

Our comparison of *a posteriori* assessments of calibration quality to the *a priori* evaluation of calibration quality based on the intrinsic fossil evidence (table 1) demonstrates that cross-validation methods do not identify accurate calibrations consistently—that is, calibrations supported on the basis of independent (palaeontological, phylogenetic and geological) evidence. In addition, the evaluation of the available fossil evidence *a priori* using best practices [30] led to a substantial revision of the age constraints for many nodes.

The advantage of implementing the cross-validation approach within the Bayesian framework is that it can account for the expected probability that the age of a node may be considerably older than its first appearance in the fossil record (or any specified minimum age constraint). None of the revised calibrations [27] were identified as statistically inconsistent through the cross-validation methods [26,32] implemented in this study. This may reflect the fact that we considered the mean estimates relative to minima, or minimum–maximum divergence time priors. It is much easier for divergence time estimates to be compatible with broad constraints than with precise node ages. If consistency is a desirable quality in a suite of calibrations, it could be argued that the penalty for achieving this quality is a loss of precision over the age of component nodes since the calibrations are ultimately more accurate but less precise. Though expert evaluation of palaeontological evidence may be best practice, perhaps a less conservative approach to evaluating fossil evidence might result in more precise calibration constraints. Hence, *a posteriori* approaches, including the cross-validation family of methods, may be an appealing alternative to wrestling with the complexities of deriving a temporal calibration from fossil, phylogenetic, stratigraphic and geochronological data. This appeal is demonstrated by the continued development (e.g. [18,20,25,32]) and application (e.g. [45–47]) of *a posteriori* methods.

Regardless, our analyses highlight the fact that the effective calibrations employed in divergence time estimation invariably differ from those specified by the user (figure 3). Consequently, different combinations of calibrations, in combination with the tree prior, will produce different joint time prior constructs—this occurs regardless of the (non-uniform or uniform) prior probability densities employed, or the approach used (directly or indirectly) to specify the (soft) maximum constraints. Thus, the manner in which a given calibration is implemented in the estimation of divergence times is not equivalent if it is employed alone or in combination with others. Furthermore, because different calibration priors have a material impact on the posteriors, consistency among either the effective priors or posteriors is not a reliable means of evaluating the relative accuracy of calibrations.

Cross-validation methods share the same implicit expectation that the influence of a single calibration on a molecular clock analysis is the same regardless of whether it is employed alone or in combination with a suite of other calibrations. Our results demonstrate that this expectation is not met since the effective time prior for any one node is not the same as the user-specified calibration, and the effective time prior differs depending upon its precise temporal and topological relationship to other calibrations. This observation calls into question the entire approach of the cross-validation family of methods for evaluating calibration through consistency, regardless of whether consistency is perceived to be an appropriate quality of a set of calibrations. Thus, cross-validation approaches to assessing the quality of calibrations based on consistency cannot be considered a reliable means of establishing accuracy, not merely because they are biologically questionable [48], but because they are flawed, both logically and methodologically.

Evaluating calibrations *a priori* places emphasis on palaeontological accuracy. At the very least, fossil minima should postdate divergence events and fossil maxima predate divergence events. Our results show that there should be no alternative to the careful evaluation of fossil evidence, in terms of comparative anatomy, phylogenetic affinity, stratigraphic occurrence and its geochronological interpretation. There can be no justification for using calibrations that are contradicted by this independent body of evidence. However, we also show that the best efforts of field palaeontologists, comparative anatomists, phylogeneticists, biostratigraphers and geochronologists may be of moot significance if carefully researched calibrations are not implemented (rather than merely specified) in molecular clock analyses. At the very least, it should be a basic requirement of every molecular clock analysis that the effective time priors are evaluated in comparison to the specified time priors by first running the analysis without sequence data [13,35]. Ultimately, it is important only that the effective time priors reflect accurately the palaeontological constraints on divergence time estimation.

## 5. Conclusion

Bayesian posterior estimates of divergence times are extremely sensitive to the time priors. We have demonstrated that slight changes in the specification of the prior probabilities have an adverse impact on posterior time estimates. In addition, we have shown that *a posteriori* approaches of assessing calibration quality can be used to explore qualitatively the relationship between minimum and maximum constraints and the putative time of divergence, but do not provide justification for the removal of any calibrations. However, we have also demonstrated that *a posteriori* methods which rely on cross-validation are incoherent since they rely on the implicit assumption that the performance of each calibration is the same regardless of whether it is employed alone or in combination with others. This assumption is violated within the Bayesian framework because the effective calibrations employed in the joint estimation of divergence times are never the same as the user-specified calibrations when more than one calibration is employed. The effective time priors always depend on the temporal and topological relationship among all calibrations included in the analysis. Although *a priori* justification generates calibrations that are based on all available evidence, which are consequently superior in terms of accuracy, they are not immune to the effects of establishing the joint time prior. Every molecular clock study should consider carefully the disparity between the specified and effective priors. This phenomenon has broad implications for any study that relies on the accurate estimation of evolutionary rates and times. Our results also underscore the need to consider simultaneously the multifaceted issues associated with calibration, such as the nature of the diverse data on which the calibrations are based and the ability of existing molecular clock methods to effectively represent these constraints.

Finally, best practice *a priori* protocols for establishing calibrations should not remain static. Recent methodological developments in approaches to calibration require additional types of palaeontological data, such as tip calibration using fossils as terminal taxa [49], or probabilistic approaches to constraining divergence times based on the distribution of stratigraphic occurrences [37,39]. These methods hold great promise for the development of increasingly accurate and precise evolutionary timescales for groups with a good fossil records and maybe even for entirely extinct lineages (e.g. [50]). However, for lineages with little or no fossil record—those groups for which the molecular clock was established—these novel calibration methods cannot be applied. Consequently, node-based calibrations will continue to play an important role in molecular dating. As we have demonstrated, establishing accurate constraints should not rely on *a posteriori* methods, and so node-based calibrations established using *a priori* methods will remain especially significant for groups for whom the molecular clock is the only means of establishing a reliable timescale.

## Supplementary Material

ESM

## Supplementary Material

Supplementary Table 1

## Data accessibility

Data available from the Dryad Digital Repository: http://doi.org/10.5061/dryad.j8776.
