# Supplementary material for: Calibration uncertainty in molecular dating analyses: there is no substitute for the prior evaluation of time priors
Source: Proc Biol Sci. 2015 Jan 7;282(1798):20141013. doi: 10.1098/rspb.2014.1013 (PMC4262156; doi:10.1098/rspb.2014.1013)
Supplement: ESM [file rspb20141013supp1.docx]

**Electronic supplementary material**

**1. Supplementary materials and methods**

**(a) Molecular data and alignments**

We used a molecular dataset of 23 taxa representing all major lineages of extant turtles (Testudines) that has been used previously to evaluate the impact of palaeontological evidence in molecular clock studies [1-4]. The dataset is comprised of the mitochondrial gene cytochrome b (cytB), the nuclear gene recombinase activating gene 1 (RAG1), and the nuclear intron R35 (RM35). Sequences were downloaded and aligned as per the protocols outlined in [5]. The matrix contains 4613 characters.

**(b) Topology**

We did not examine the impact of topological uncertainty on molecular clock estimates as part of this study (but see [1, 6]). We adopted an emerging consensus from molecular phylogenies of extant turtles (e.g., [7-10]), assuming monophyly of Cryptodira and Pleurodira (e.g., [11-13]). The resulting topology (figure 3) was kept constant through all analyses.

**(c) Model selection and partition choice**

We used Bayes factors to select the best partition scheme [14]. The data was partitioned into six alternative partition schemes, applying separate models to each partition, as selected by the AIC in MrModelTest 2.2 [15]. Bayesian phylogenetic analysis was performed using the MPI version of MrBayes 3.1.2 [16-18], unlinking model parameters between partitions, with two parallel runs of five million generations, sampling every 100 steps and discarding the first 15 000 trees as burn-in. We constrained node 14 (Chelydroidea) and node 18 (Emydidae–*P. megacephalum*) to be monophyletic across all partition schemes. Convergence was assessed using standard diagnostics (effective sample size and standard deviation of split frequencies) and trace plots were assessed visually in Tracer 1.5 [19]. The harmonic mean was estimated using the “sump” command in MrBayes. Bayes Factors were calculated as the ratio of the harmonic means of the likelihood values of the MCMC samples of the two competing models, which approximates the ratio of their marginal likelihoods. The natural logarithm of this ratio was then doubled and a cutoff value of ten or more was applied following [14]. The most complex partition strategy was preferred.

**(d) MCMCTREE settings – assessing the impact of arbitrary non-uniform priors and cross-validation**

Due to computational limitations and the large number of partitions, we used the approximate likelihood calculation implemented in MCMCTREE [20] (PAML 4 package, [21]). Maximum likelihood estimates of branch lengths were estimated using BASEML [21]. Different substitution models cannot be applied to individual partitions in MCMCTREE but the model parameters can be unlinked across partitions. Therefore, we used the complex GTR + ᒥ model with 5 gamma rate categories which, of the models available in MCMCTREE, best describes the substitution process in all but one partition according to the AIC, for the likelihood estimation of branch lengths. Molecular clock estimates were obtained using the independent rates model, with the gamma prior on the overall substitution rate (*rgene_gamma*) and rate-drift parameter (*sigma2_gamma*) specified with respective means and standard deviations *G* (0.041, 0.041) and *G* (0.2, 0.2). Two independent runs were performed, each consisting of 5 million iterations, discarding the first 500,000 generations as burn-in and sampling every 100th generation, resulting in a total of 45,000 samples post burn-in. All prior and posterior output was examined in Tracer. For the cross-validation analysis, we implemented the birth (λ=1), death (μ=1), sampling fraction (ρ=0) prior on times (*BDSparas*). This produces a diffuse prior to describe the distribution of divergence times for the nodes lacking calibrations during each round of cross-validation.

**(e) Implementing arbitrary non-uniform priors**

We explored the use of arbitrary non-uniform calibration priors on divergence time estimation using the truncated Cauchy distribution, applied to the minimum calibration bound in MCMCTREE [22]. Increasing the uncertainty associated with fossil minima by permuting the parameters of a non-uniform density is equivalent to increasing prior probability that the divergence time may be older than the age of the minimum. The shape of the truncated Cauchy distribution can be manipulated easily by changing the location (*p*) and scale (*c*) parameters. The location parameter changes the position of the peak in prior probability and the scale parameter controls the amplitude of the distribution, while the distribution is truncated at the minimum bound. We explored the use of two different values of *c* (0.1, 0.5) and 4 different values of *p* (0.2, 0.5, 1, 2) to reflect different probabilities of divergence timing relative to our minimum constraints. Note the truncated Cauchy distribution cannot be applied to the root of the tree in MCMCTREE, and so we retained a uniform prior at the root, with a hard minimum and soft maximum constraint corresponding to the fossil-based calibration available for the age of this node [5].

**(f) Statistical analysis of cross-validation output**

We used the average difference (D*_x_*) and the sum of squared differences (SS) between the mean posterior molecular estimates and the minimum fossil age estimates to assess the disparity between calibrations. For each calibration being assessed, the D*_x_* and SS values are based on the differences between the mean estimates and the calibrations of all other nodes. This procedure has been outlined in detail elsewhere [4, 23]. We then recalculated D*_x_* and SS to reflect inconsistency when the entire calibration span is considered, such that D*_x_* represents the difference between the mean molecular estimates and the minimum or maximum fossil age estimates [24]. Differences were expressed in terms of millions of years; mean estimates that are older than the maximum constraints are positive, while age estimates that are younger than the minimum are negative, estimates that fall between the minimum and maximum constraints are equal to zero. A one-tailed F-test was used to determine whether the exclusion of the most inconsistent fossils would result in a significant difference in the average squared deviation (*s*) of estimates, therefore minimizing the difference between the divergence time estimates and the fossil constraints [4, 23].

We could alternatively have measured consistency based on the peak in posterior probability, the 95% credibility intervals, or the entire posterior distribution. However, the posterior mean is mathematically straightforward to calculate, easy to incorporate into existing formulae for cross-validation, and we are confident that the means satisfactorily capture the overall trends.

**(d) MCMCTREE settings – comparison between BEAST and MCMCTREE**

Prior and posterior estimates of divergence times were obtained using BEAST 1.6.1 [19, 25] and MCMCTREE [26, 27]. Where possible, parameters were chosen such that differences between BEAST and MCMCTREE were minimized. Consequently we implemented the full likelihood calculation in MCMCTREE, rather than the approximate likelihood calculation implemented in the above analyses. However, this implementation is only really practical in MCMCTREE for very small species trees [21], and this made our analysis prohibitively slow. To mitigate this problem we removed the R35 locus from this section of analysis. Removing this relatively small locus had little impact on the estimates. For the Bayesian estimation of substitution parameters, the most complex model that can be implemented in MCMCTREE is the HKY model. We therefore used the HKY substitution model in both programs. We examined the use of more complex substitution models in BEAST to determine their impact on the Bayesian estimation of substitution rates and age estimates and found that this did not have a substantial impact on the results. For the gamma priors on alpha and kappa in MCMCTREE we used a mean and standard deviation of *G*(1, 1) and *G*(3, 1.73), respectively. All other parameters and chain settings were implemented as above.

We used the uncorrelated lognormal relaxed clock (independent rates) model implemented in BEAST (fixed topology mode) [25], with the clock model and substitution model unlinked across partitions. A gamma prior was used to specify the overall substitution rate (*ucld.mean*), as in MCMCTREE, and we used a gamma prior *G*(0.45, 0.45) to specify the parameter that describes how rates vary across the tree (*ucld.stdev*). In BEAST this prior specifies the standard deviation of the lognormal distribution, whereas the equivalent parameter in MCMCTREE (*sigma2_gamma*) specifies the variance of the lognormal distribution. We set the mean of the standard deviation (*ucld.stdev*) equal to the square root of the mean of the variance (*sigma2_rgene*). It should be noted that the mean and standard deviation are specified differently in BEAST and MCMCTREE; mean = αβ and α/β, respectively. The priors used to specify alpha and kappa were equivalent to those used in MCMCTREE. Because we place a calibration on every node, the tree priors used to describe the density of ages at non-calibrated nodes in MCMCTREE is not relevant here. However, BEAST generates the joint time prior by multiplying the calibration priors with the density of times specified by the tree prior [28]. We implemented the birth-death tree prior [29] available in BEAST, and fixed the values of λ and μ to 1 and 0.5, respectively. In BEAST both minimum and maximum bounds are hard, whereas in MCMCTREE we applied hard minimum and soft maximum constraints, as above.

Two independent runs were performed, each consisting of 100 million iterations, discarding the first 25 million generations as burn-in and sampling every 1500 generations, resulting in a total of 50,000 samples post burn-in. All analyses were run twice to ensure convergence and the output from independent runs was examined in Tracer. Input files for the two Bayesian programs have been deposited on DRYAD.

**References**

1. Dornburg A, Beaulieu JM, Oliver JC, Near TJ. 2011 Integrating fossil preservation biases in the selection of calibrations for molecular divergence time estimation. *Syst. Biol.* **60**, 519–527. (doi:10.1093/Sysbio/Syr019)

2. Heath TA. 2012 A hierarchical Bayesian model for calibrating estimates of species divergence times. *Syst. Biol.* **61**, 793–809. (doi:10.1093/Sysbio/Sys032)

3. Marshall CR. 2008 A simple method for bracketing absolute divergence times on molecular phylogenies using multiple fossil calibration points. *Am. Nat.* **171**, 726–742. (doi:10.1086/587523)

4. Near TJ, Meylan PA, Shaffer HB. 2005 Assessing concordance of fossil calibration points in molecular clock studies: An example using turtles. *Am. Nat.* **165**, 137–146. (doi:10.1086/427734)

5. Joyce WG, Parham JF, Lyson TR, Warnock RCM, Donoghue PCJ. 2013 A divergence dating analysis of turtles using fossil calibrations: an example of best practices. *J Paleontol.* **87**, 612–634. (doi:10.1666/12-149)

6. Sterli J, Pol D, Laurin M. 2013 Incorporating phylogenetic uncertainty on phylogeny-based palaeontological dating and the timing of turtle diversification. *Cladistics* **29,** 233–246. (doi:10.1111/j.1096-0031.2012.00425.x)

7. Barley AJ, Spinks PQ, Thomson RC, Shaffer HB. 2010 Fourteen nuclear genes provide phylogenetic resolution for difficult nodes in the turtle tree of life. *Mol. Phylogenet. Evol.* **55**, 1189–1194. (doi:10.1016/J.Ympev.2009.11.005)

8. Fujita MK, Engstrom TN, Starkey DE, Shaffer HB. 2004 Turtle phylogeny: insights from a novel nuclear intron. *Mol. Phylogenet. Evol.* **31**, 1031–1040. (doi:Doi 10.1016/J.Ympev.2003.09.016)

9. Krenz JG, Naylor GJP, Shaffer HB, Janzen FJ. 2005 Molecular phylogenetics and evolution of turtles. *Mol*. *Phylogenet*. *Evol*. **37**, 178–191. (doi:10.1016/J.Ympev.2005.04.027)

10. Parham JF, Feldman CR, Boore JL. 2006 The complete mitochondrial genome of the enigmatic bigheaded turtle (*Platysternon*): description of unusual genomic features and the reconciliation of phylogenetic hypotheses based on mitochondrial and nuclear DNA. *BMC Evol. Biol.* **6**, 1–11. (doi:10.1186/1471-2148-6-11)

11. Gaffney ES, Tong HY, Meylan PA. 2006 Evolution of the side-necked turtles: The families Bothremydidae, Euraxemydidae, and Araripemydidae. *Bull. Am. Mus. Nat. Hist.* **300**, 1-318.

12. Hirayama R, Brinkman DB, Danilov IG. 2000 Distribution and biogeography of non-marine Cretaceous turtles. *Russ. J. Herpetol.* **7**, 181–198.

13. Joyce WG. 2007 Phylogenetic relationships of Mesozoic turtles. *Bull. Peabody Mus. Nat. Hist.* **48**, 3–102. (doi:10.3374/0079-032x(2007)48[3:promt]2.0.co;2)

14. Kass RE, Raftery AE. 1995 Bayes Factors. *J. Am. Stat. Assoc.* **90**, 773–795. (doi:Doi 10.2307/2291091)

15. Nylander JAA. 2004 MrModeltest v2. Program distributed by the author. Evolutionary Biology Centre, Uppsala University.

16. Altekar G, Dwarkadas S, Huelsenbeck JP, Ronquist F. 2004 Parallel Metropolis coupled Markov chain Monte Carlo for Bayesian phylogenetic inference. *Bioinformatics* **20**, 407–415. (doi:10.1093/bioinformatics/btg427)

17. Huelsenbeck JP, Ronquist F. 2001 MrBayes: Bayesian inference of phylogenetic trees. *Bioinformatics* **17**, 754–755.

18. Ronquist F, Huelsenbeck JP. 2003 MrBayes 3: Bayesian phylogenetic inference under mixed models. *Bioinformatics* **19**, 1572–1574.

19. Drummond AJ, Rambaut A. 2007 BEAST: Bayesian evolutionary analysis by sampling trees. *BMC Evol. Biol.* **7/214.** (doi:10.1186/1471-2148-7-214)

20. dos Reis M, Yang ZH. 2011 Approximate likelihood calculation on a phylogeny for Bayesian estimation of divergence times. *Mol. Biol. Evol.* **28**, 2161–2172. (doi:10.1093/Molbev/Msr045)

21. Yang ZH. 2007 PAML 4: Phylogenetic Analysis by Maximum Likelihood. *Mol. Biol. Evol.* **24**, 1586–1591. (doi:10.1093/Molbev/Msm088)

22. Inoue J, Donoghue PCJ, Yang ZH. 2010 The impact of the representation of fossil calibrations on Bayesian estimation of species divergence times. *Syst. Biol.* **59**, 74–89. (doi:10.1093/Sysbio/Syp096)

23. Near TJ, Bolnick DI, Wainwright PC. 2005 Fossil calibrations and molecular divergence time estimates in centrarchid fishes (Teleostei: Centrarchidae). *Evolution* **59**, 1768–1782.

24. Clarke JT, Warnock RCM, Donoghue PCJ. 2011 Establishing a time-scale for plant evolution. *New Phytol.* **192**, 266–301. (doi:10.1111/J.1469-8137.2011.03794.X)

25. Drummond AJ, Ho SYW, Phillips MJ, Rambaut A. 2006 Relaxed phylogenetics and dating with confidence. *Plos Biol.* **4**, 699–710. (doi:10.1371/Journal.Pbio.0040088)

26. Rannala B, Yang ZH. 2007 Inferring speciation times under an episodic molecular clock. *Syst. Biol.* **56**, 453–466. (doi:10.1080/10635150701420643)

27. Yang ZH, Rannala B. 2006 Bayesian estimation of species divergence times under a molecular clock using multiple fossil calibrations with soft bounds. *Mol. Biol. Evol.* **23**, 212–226. (doi:10.1093/Molbev/Msj024)

28. Heled J, Drummond AJ. 2012 Calibrated tree priors for relaxed phylogenetics and divergence time estimation. *Syst. Biol.* **61**, 138–149. (doi:10.1093/Sysbio/Syr087)

29. Gernhard T. 2008 The conditioned reconstructed process. *J. Theor. Biol.* **253**, 769–778.
