# Supplementary material for: Calibration uncertainty in molecular dating analyses: there is no substitute for the prior evaluation of time priors
Source: Proc Biol Sci. 2015 Jan 7;282(1798):20141013. doi: 10.1098/rspb.2014.1013 (PMC4262156; doi:10.1098/rspb.2014.1013)
Supplement: Supplementary Table 1 [file rspb20141013supp2.docx]

**Supplementary Table S1.** Prior and posterior age estimates (mean and 95% credibility limits, given in MYA) obtained using non-uniform and uniform priors in MCMCTREE.

| Node ID ^a^ | Mean | Lower and upper 95% intervals | | Mean | Lower and upper 95% intervals | | Mean | Lower and upper 95% intervals | | Mean | Lower and upper 95% intervals | | Mean | Lower and upper 95% intervals | | Mean | Lower and upper 95% intervals | | Mean | Lower and upper 95% intervals | | Mean | Lower and upper 95% intervals | |
| --- | --- | --- | --- | --- | --- | --- | --- | --- | --- | --- | --- | --- | --- | --- | --- | --- | --- | --- | --- | --- | --- | --- | --- | --- |
| *Prior age estimates* | | | |  |  |  |  |  |  |  |  |  |  |  |  |  |  |  |  |  |  |  |  |  |
|  | *p = 0.1, c = 0.2*^b^ | | | *p = 0.1, c = 0.5* | | | *p = 0.1, c = 1* | | | *p = 0.1, c = 2* | | | *p = 0.5, c = 0.2* | | | *p = 0.5, c = 0.5* | | | *p = 0.5, c = 1* | | | *p = 0.5, c = 2* | | |
|  |  |  |  |  |  |  |  |  |  |  |  |  |  |  |  |  |  |  |  |  |  |  |  |  |
| 1 | 213 | 161 | 252 | 224 | 173 | 254 | 232 | 187 | 255 | 239 | 202 | 256 | 226 | 185 | 253 | 231 | 189 | 255 | 236 | 197 | 255 | 240 | 206 | 257 |
| 2 | 142 | 113 | 205 | 164 | 116 | 230 | 182 | 121 | 240 | 195 | 128 | 246 | 173 | 135 | 222 | 182 | 128 | 237 | 2 | 142 | 113 | 205 | 164 | 116 |
| 3 | 110 | 94 | 143 | 121 | 94 | 178 | 132 | 94 | 201 | 142 | 95 | 217 | 136 | 99 | 174 | 136 | 96 | 194 | 141 | 95 | 209 | 145 | 95 | 219 |
| 4 | 8 | 5 | 23 | 12 | 5 | 47 | 16 | 6 | 67 | 23 | 6 | 92 | 9 | 6 | 22 | 12 | 6 | 41 | 16 | 6 | 64 | 23 | 6 | 93 |
| 5 | 83 | 66 | 126 | 96 | 66 | 160 | 111 | 67 | 191 | 127 | 69 | 211 | 102 | 71 | 150 | 109 | 68 | 176 | 119 | 68 | 197 | 131 | 69 | 214 |
| 6 | 17 | 12 | 41 | 22 | 12 | 62 | 29 | 12 | 83 | 38 | 12 | 110 | 20 | 13 | 41 | 23 | 12 | 64 | 30 | 12 | 85 | 39 | 13 | 112 |
| 7 | 19 | 14 | 45 | 24 | 14 | 65 | 32 | 14 | 86 | 42 | 14 | 116 | 23 | 15 | 47 | 26 | 14 | 67 | 33 | 14 | 89 | 43 | 15 | 116 |
| 8 | 164 | 132 | 220 | 187 | 141 | 239 | 205 | 155 | 246 | 218 | 172 | 249 | 195 | 159 | 234 | 202 | 157 | 243 | 212 | 165 | 247 | 221 | 176 | 250 |
| 9 | 141 | 125 | 174 | 152 | 125 | 204 | 163 | 126 | 220 | 171 | 126 | 230 | 169 | 129 | 206 | 167 | 127 | 218 | 169 | 126 | 226 | 173 | 126 | 232 |
| 10 | 25 | 18 | 62 | 33 | 18 | 99 | 43 | 18 | 125 | 56 | 18 | 149 | 29 | 19 | 63 | 35 | 18 | 98 | 44 | 18 | 125 | 57 | 19 | 152 |
| 11 | 116 | 91 | 163 | 145 | 101 | 206 | 172 | 119 | 226 | 194 | 140 | 238 | 144 | 115 | 190 | 162 | 119 | 215 | 181 | 131 | 231 | 198 | 146 | 239 |
| 12 | 95 | 77 | 127 | 116 | 84 | 168 | 140 | 94 | 198 | 163 | 107 | 219 | 119 | 98 | 151 | 133 | 98 | 180 | 151 | 103 | 205 | 169 | 112 | 222 |
| 13 | 60 | 49 | 87 | 70 | 49 | 114 | 82 | 50 | 143 | 97 | 51 | 173 | 75 | 52 | 107 | 80 | 51 | 127 | 89 | 51 | 152 | 101 | 51 | 177 |
| 14 | 84 | 73 | 104 | 97 | 75 | 136 | 115 | 78 | 168 | 134 | 84 | 194 | 106 | 88 | 127 | 113 | 84 | 152 | 125 | 84 | 176 | 139 | 87 | 199 |
| 15 | 77 | 70 | 90 | 84 | 71 | 112 | 94 | 71 | 138 | 107 | 72 | 164 | 95 | 75 | 113 | 96 | 72 | 128 | 102 | 72 | 149 | 111 | 73 | 170 |
| 16 | 62 | 53 | 76 | 66 | 53 | 90 | 72 | 54 | 108 | 78 | 54 | 128 | 76 | 56 | 96 | 75 | 54 | 104 | 77 | 54 | 117 | 81 | 54 | 137 |
| 17 | 72 | 54 | 111 | 92 | 58 | 151 | 117 | 66 | 185 | 144 | 79 | 211 | 88 | 66 | 132 | 103 | 66 | 163 | 124 | 72 | 193 | 149 | 84 | 214 |
| 18 | 45 | 34 | 68 | 56 | 36 | 97 | 71 | 38 | 131 | 91 | 43 | 164 | 55 | 42 | 80 | 63 | 40 | 104 | 76 | 41 | 134 | 95 | 44 | 168 |
| 19 | 37 | 32 | 47 | 41 | 32 | 62 | 48 | 33 | 84 | 58 | 33 | 113 | 45 | 33 | 58 | 46 | 33 | 70 | 51 | 33 | 90 | 60 | 33 | 117 |
| 20 | 4 | 3 | 12 | 6 | 3 | 21 | 8 | 3 | 30 | 12 | 3 | 41 | 5 | 3 | 12 | 6 | 3 | 20 | 8 | 3 | 30 | 12 | 3 | 40 |
| 21 | 58 | 51 | 76 | 66 | 51 | 101 | 77 | 51 | 132 | 92 | 52 | 165 | 71 | 53 | 92 | 75 | 52 | 114 | 83 | 52 | 140 | 96 | 52 | 170 |
| 22 | 8 | 5 | 20 | 11 | 5 | 35 | 14 | 6 | 50 | 20 | 6 | 68 | 9 | 6 | 20 | 11 | 6 | 34 | 15 | 6 | 50 | 21 | 6 | 69 |
|  |  |  |  |  |  |  |  |  |  |  |  |  |  |  |  |  |  |  |  |  |  |  |  |  |
| *Posterior age estimates* | | | |  |  |  |  |  |  |  |  |  |  |  |  |  |  |  |  |  |  |  |  |  |
|  |  |  |  |  |  |  |  |  |  |  |  |  |  |  |  |  |  |  |  |  |  |  |  |  |
| 1 | 215 | 180 | 249 | 236 | 204 | 255 | 243 | 217 | 257 | 246 | 224 | 258 | 240 | 216 | 255 | 243 | 220 | 257 | 245 | 223 | 258 | 246 | 226 | 259 |
| 2 | 164 | 132 | 205 | 185 | 150 | 223 | 195 | 161 | 230 | 200 | 167 | 235 | 186 | 161 | 217 | 194 | 163 | 226 | 198 | 165 | 233 | 201 | 168 | 236 |
| 3 | 117 | 96 | 150 | 134 | 103 | 169 | 142 | 109 | 177 | 147 | 114 | 182 | 138 | 113 | 163 | 142 | 113 | 173 | 146 | 114 | 180 | 148 | 115 | 183 |
| 4 | 35 | 19 | 54 | 39 | 22 | 60 | 41 | 23 | 64 | 43 | 25 | 66 | 40 | 22 | 61 | 41 | 23 | 63 | 42 | 23 | 65 | 43 | 25 | 66 |
| 5 | 93 | 71 | 124 | 108 | 81 | 139 | 116 | 88 | 148 | 122 | 93 | 154 | 107 | 87 | 134 | 114 | 88 | 144 | 119 | 91 | 150 | 123 | 94 | 155 |
| 6 | 76 | 52 | 105 | 88 | 61 | 119 | 95 | 66 | 127 | 99 | 70 | 133 | 87 | 63 | 115 | 93 | 65 | 123 | 97 | 67 | 129 | 100 | 70 | 133 |
| 7 | 40 | 20 | 63 | 47 | 26 | 72 | 51 | 30 | 77 | 55 | 33 | 82 | 44 | 22 | 70 | 48 | 27 | 75 | 52 | 30 | 78 | 55 | 33 | 82 |
| 8 | 189 | 155 | 230 | 210 | 173 | 243 | 217 | 183 | 246 | 220 | 187 | 248 | 215 | 186 | 243 | 218 | 187 | 246 | 220 | 187 | 248 | 221 | 188 | 248 |
| 9 | 157 | 129 | 195 | 176 | 142 | 210 | 183 | 150 | 215 | 187 | 154 | 218 | 182 | 155 | 209 | 185 | 153 | 215 | 186 | 153 | 217 | 187 | 154 | 218 |
| 10 | 96 | 69 | 128 | 108 | 78 | 140 | 113 | 83 | 145 | 116 | 85 | 148 | 111 | 83 | 141 | 113 | 83 | 145 | 115 | 84 | 147 | 116 | 85 | 148 |
| 11 | 100 | 90 | 115 | 111 | 95 | 130 | 118 | 100 | 139 | 122 | 103 | 146 | 120 | 106 | 136 | 121 | 104 | 141 | 123 | 103 | 145 | 124 | 104 | 148 |
| 12 | 95 | 85 | 109 | 106 | 91 | 124 | 112 | 95 | 133 | 116 | 97 | 139 | 114 | 101 | 130 | 115 | 99 | 134 | 117 | 98 | 138 | 118 | 99 | 141 |
| 13 | 56 | 49 | 69 | 61 | 49 | 79 | 65 | 50 | 86 | 67 | 50 | 90 | 69 | 53 | 84 | 67 | 51 | 87 | 67 | 50 | 90 | 68 | 50 | 92 |
| 14 | 93 | 82 | 107 | 102 | 88 | 120 | 109 | 92 | 129 | 113 | 94 | 135 | 111 | 98 | 126 | 112 | 95 | 130 | 113 | 95 | 134 | 115 | 95 | 137 |
| 15 | 77 | 70 | 88 | 82 | 71 | 97 | 86 | 72 | 104 | 89 | 73 | 108 | 90 | 75 | 105 | 89 | 74 | 106 | 89 | 74 | 108 | 90 | 74 | 109 |
| 16 | 56 | 53 | 62 | 56 | 53 | 65 | 57 | 53 | 67 | 57 | 53 | 68 | 59 | 53 | 73 | 58 | 53 | 69 | 58 | 53 | 69 | 58 | 53 | 69 |
| 17 | 66 | 56 | 77 | 71 | 59 | 85 | 74 | 62 | 90 | 77 | 63 | 95 | 75 | 63 | 88 | 75 | 62 | 91 | 77 | 63 | 93 | 78 | 64 | 97 |
| 18 | 62 | 51 | 74 | 67 | 54 | 81 | 70 | 56 | 87 | 73 | 58 | 91 | 70 | 56 | 84 | 71 | 57 | 87 | 72 | 58 | 90 | 74 | 59 | 93 |
| 19 | 36 | 32 | 43 | 37 | 32 | 48 | 38 | 32 | 51 | 40 | 32 | 54 | 41 | 33 | 52 | 40 | 32 | 52 | 40 | 32 | 53 | 40 | 32 | 55 |
| 20 | 6 | 3 | 13 | 8 | 3 | 15 | 9 | 4 | 17 | 10 | 4 | 18 | 6 | 4 | 14 | 8 | 4 | 16 | 9 | 4 | 17 | 10 | 4 | 18 |
| 21 | 53 | 50 | 60 | 54 | 50 | 63 | 55 | 50 | 66 | 56 | 50 | 69 | 57 | 51 | 69 | 56 | 50 | 68 | 56 | 50 | 68 | 56 | 50 | 70 |
| 22 | 36 | 19 | 49 | 37 | 21 | 51 | 38 | 22 | 53 | 39 | 23 | 55 | 38 | 20 | 55 | 38 | 21 | 54 | 38 | 22 | 54 | 39 | 23 | 55 |

**Continued on the next page.**

**^a^** Node ID corresponds to those shown Fig. 3.

**^b^** *p* and *c* refer to the location and scale parameters of the truncated Cauchy distribution.

**Supplementary Table S1 continued.** Prior and posterior age estimates (mean and 95% credibility limits, given in MYA) obtained using non-uniform and uniform priors in MCMCTREE.

| Node ID^a^ | Mean | Lower and upper 95% intervals | |
| --- | --- | --- | --- |
| *Prior age estimates* | | | |
|  | *Uniform priors* | | |
|  |  |  |  |
| 1 | 214 | 170 | 254 |
| 2 | 148 | 121 | 167 |
| 3 | 121 | 93 | 147 |
| 4 | 63 | 5 | 121 |
| 5 | 116 | 74 | 151 |
| 6 | 64 | 12 | 122 |
| 7 | 64 | 13 | 122 |
| 8 | 174 | 146 | 202 |
| 9 | 148 | 124 | 173 |
| 10 | 81 | 17 | 140 |
| 11 | 140 | 120 | 154 |
| 12 | 126 | 99 | 150 |
| 13 | 87 | 48 | 128 |
| 14 | 110 | 79 | 139 |
| 15 | 94 | 70 | 122 |
| 16 | 74 | 53 | 101 |
| 17 | 112 | 78 | 146 |
| 18 | 77 | 45 | 103 |
| 19 | 54 | 32 | 85 |
| 20 | 19 | 3 | 33 |
| 21 | 75 | 50 | 99 |
| 22 | 35 | 5 | 63 |
|  |  |  |  |
| *Posterior age estimates* | | | |
|  |  |  |  |
| 1 | 219 | 196 | 243 |
| 2 | 161 | 148 | 170 |
| 3 | 121 | 100 | 141 |
| 4 | 42 | 24 | 61 |
| 5 | 110 | 87 | 133 |
| 6 | 91 | 67 | 116 |
| 7 | 54 | 33 | 77 |
| 8 | 193 | 176 | 206 |
| 9 | 163 | 144 | 179 |
| 10 | 104 | 80 | 128 |
| 11 | 116 | 97 | 135 |
| 12 | 110 | 92 | 129 |
| 13 | 65 | 48 | 83 |
| 14 | 107 | 90 | 126 |
| 15 | 85 | 70 | 99 |
| 16 | 57 | 53 | 64 |
| 17 | 76 | 61 | 91 |
| 18 | 72 | 57 | 89 |
| 19 | 39 | 32 | 51 |
| 20 | 12 | 4 | 19 |
| 21 | 56 | 50 | 66 |
| 22 | 42 | 27 | 56 |

^a^ Node ID corresponds to those shown Fig 3.
